# Resting-State Neuroimaging and Neuropsychological Findings in Opioid Use Disorder during Abstinence: A Review

**DOI:** 10.3389/fnhum.2017.00169

**Published:** 2017-04-06

**Authors:** Hada Fong-ha Ieong, Zhen Yuan

**Affiliations:** Bioimaging Core, Faculty of Health Sciences, University of MacauTaipa, Macau

**Keywords:** heroin, opioid, electroencephalography, functional magnetic resonance imaging, neuropsychological tests, resting-state functional connectivity, cognition, addiction

## Abstract

Dependence to opiates, including illicit heroin and prescription pain killers, and treatment of the opioid use disorder (OUD) have been longstanding problems over the world. Despite intense efforts to scientific investigation and public health care, treatment outcomes have not significantly improved for the past 50 years. One reason behind the continuing use of heroin worldwide despite such efforts is its highly addictive nature. Brain imaging studies over the past two decades have made significant contribution to the understanding of the addictive properties as to be due in part to biological processes, specifically those in the brain structure and function. Moreover, traditional clinical neuropsychology studies also contribute to the account in part for the treatment-refractory nature of the drug abuse. However, there is a gap between those studies, and the rates of relapse are still high. Thus, a multidisciplinary approach is needed to understand the fundamental neural mechanism of OUD. How does the brain of an OUD patient functionally and cognitively differ from others? This brief review is to compare and contrast the current literature on non-invasive resting state neuroimaging and clinical neuropsychological studies with the focus on the abstinence stage in OUD. The results show as follow:

1. Brain connectivity strength in the reward system, dysregulation of circuits associated with emotion and stress, enhanced beta and alpha power activity, and high impulsivity are induced by OUD.
2. Some recovery signs in cognition are demonstrated in OUD subjects after prolonged abstinence, but not in the subjects undergoing methadone treatment.
3. Normalization in the composition of brain oscillations especially in the temporal region is induced and restored by methadone treatment in roughly 6 months in mean duration for OUDs having a mean opioid-use history of 10 years.

We hope that the review provides valuable implications for clinical research and practice and paves a new insight into the future path to the identification of potential biomarkers and clinical outcome predictors in OUD in the domains of brain regions, functions, and behaviors.

## Introduction

Among the abused substances, opiates including illicit heroin and prescription opiates are one of the most frequently abused and powerfully dependent drugs. Opioid use disorder (OUD) has been associated with severe and dire global and societal consequences. Overdose is a major cause of death (Levi et al., 2012), and the prevalence of blood borne viruses, such as HIV and hepatitis C, are higher in illicit opiate users than the general population (Bart, 2012; United Nations Office on Drugs and Crime, 2014). The social burden due to OUD is tremendous but current pharmacological treatments of opioid-related conditions and psychological therapies are still insufficient to reduce the high relapse rate.

Substance use disorders (SUDs) are characterized as the compulsion of drug-seeking and drug-taking behaviors despite of harmful consequences, and it encircles a relapsing cycle of intoxication, binging, withdrawal and craving during abstinence (Goldstein and Volkow, 2011). It becomes the notion that the loss of control over drug consumption involves disruption of the reward mesocorticolimbic (MCL) circuits. However, how different substances of abuse manipulate particular brain regions within the circuits and to what extend the affected regions are related to certain cognition and behaviors need further elucidation.

Neuroimaging findings and clinical implications have provided convincing evidence that chronic opiate abuse affects the prefrontal cortex (PFC) (Parvaz et al., 2011), temporal insula and thalamus(Goldstein and Volkow, 2002), nucleus accumbens (Noel and Gratton, 1995), amygdala (Baxter et al., 2000), and sensorimotor cortices (Liu et al., 2009). However, the existing findings have not yet been sufficient to capture the integrity of the physiological events (i.e., brain regions) and psychological events (i.e., brain functions, and behaviors) in OUD. Examining how these two domains are entangled is essential to understand the fundamentals of neural mechanism aiming to improve clinical outcomes in the disorder.

Being able to remain abstinent is a positive sign of a clinical outcome (Reilly et al., 1995; Schuckit, 2016), especially for individuals with OUD of which the relapse rate has been the highest among SUDs (SAMHSA). The neural characteristics of OUD individuals regarding to the physiological and psychological events may vary in different stages of the relapsing cycle. Here we provide a review of the chronic effects of opiate focusing on OUD individuals who stayed abstinent for more than 2 weeks—when the acute withdrawal is subside. Particular emphasis is placed on the findings of two resting-state neuroimaging: (1) the high spatial resolution technique functional magnetic resonance imaging (fMRI), and (2) the high temporal resolution technique electroencephalography (EEG), as well as the evidences of neuropsychological performances.

The aim is to highlight the important aspects of integrating connectivity between brain regions, brain functions and behaviors in OUD. The joint approaches of resting-state neuroimaging and neuropsychology are suggested to increase the effectiveness of intervention during the abstinence stage for OUD long-term recovery. We hope the review make implication to the identification of potential biomarkers to prevent relapse.

## Methodological approaches

The available literature on OUD during abstinence was identified by means of regular searches in online electronic databases through the ISI WEB of Science. The scope of the current review was focused on human non-invasive fMRI and EEG resting-state neuroimaging and neuropsychological approaches to OUD studies during the abstinence period. The basic search terms included opiate, opioid, heroin, prescription opiates, morphine, dependence, addiction, abstinence. The selected neuroimaging studies used fMRI and EEG as an evaluation tool for assessing the brain activity differences among OUD subjects and control subjects. In addition to the basic search terms, the following search terms: resting state, functional connectivity, fMRI, EEG, were included. The fMRI studies related to structural connectivity were not considered in the review. The selected cognition and behavior studies on the other hand used neuropsychological assessments as an evaluation tool for assessing the performance differences between the study and control groups. In addition to the basic search terms, the following terms: cognition, task, neuropsychology, behavior, were searched. The articles from 1969 until November 2016 were included. Inclusion criteria for both neuroimaging and neuropsychological approaches for the study group were: (1) the OUD subjects meeting the standard (DSM IV axis 1, ICD-10) criteria, and (2) the subjects stayed abstinence for a minimum of 2 weeks. Because comorbidity, such as dependence to other substances, is not uncommon in OUD individuals, the concurrent medical patterns of the studies and treatment programs were documented in the review. However, studies of substances other than opiates as the primary drug of dependence were not considered in the review.

## Neuroimaging: resting-state functional connectivity (rsFC)

Functional connectivity (FC) is a statistical dependence between the time series of measured neurophysiological signals (Gerstein and Perkel, 1969; Sporns et al., 2004). The basic concept is that two locations of a brain are considered to be functionally connected if they have coherent or synchronize dynamics. And the interpretation of different measures in the neurophysiological signals depends on the type of recordings being analyzed. Current advances in assessing brain network through resting-state functional connectivity (rsFC) using fMRI may allow for such system-level assessments and using EEG may allow for cell-level assessments. For example, a statistical dependence between neural time series in hemodynamic signals measured with fMRI could offer an interpretation into neuronal communication in the system between brain regions, whereas correlations in the spiking output of neurons measured with EEG could provide an interpretation as having functional interactions between neurons—embodied in the concept of a cell assembly, proposed by Hebb (2005). Emerging evidence supports the theory that circuits synchronized at rest are constrained to known, direct or polysynaptic anatomically interconnected regions (Damoiseaux and Greicius, 2009; Greicius et al., 2009; Honey et al., 2009), thus constituting plausible functional networks (Sutherland et al., 2012). More importantly, the strength of these resting-state networks could predict both behavioral accuracy and subsequent activation of the same brain areas when performing cognitive tasks (Sutherland et al., 2012). Therefore, alteration in rsFC can potentially be helpful to assess various neuropsychiatric trajectories in SUDs. The main goal of brain activity studies in SUDs is to assess the neurophysiological changes among the individuals dependent on the substances. Over the past several decades, non-invasive neuroimaging has contributed significant evidences into the neuroplastic adaptations that resulted from chronic drug intake. This review focuses on the studies of the rsFC generated by two powerful modalities of neurophysiological imaging: fMRI and EEG.

## Resting-state functional magnetic resonance imaging (fMRI)

fMRI is a neuroimaging technique indirectly measuring brain activity by detecting neurophysiological changes in the cerebral blood flow and thus predicting neuronal activation in an area of the brain. Despite of the raising promise that rsFC may serve as a potential diagnostic tool for various mental disorders, rsFC has been applied in only a handful of opiate-related studies (Sutherland et al., 2012). Table 1 shows the summary of current findings of resting-state fMRI studies in the review.

**Table 1:** **Summary of rs-fMRI findings in opiate addiction**.

**Features of samples**								
				**Features of opiate group**				**Finding**
**Study**	**Sample size (gender; age: mean ± sd)**	**Smoking habit**	**Educational level (mean ± sd)**	**Abuse duration (mean ± sd)**	**Abstinent duration (mean ± sd)**	**Treatment program**	**Concurrent medical patterns**	
Xie et al., 2011	22 ABS (M; 33.0 ± 6.0);	-	10.9 ± 2.4 y;	6.6 ± 3.7 y	8.0 ± 2.5 w	-	-	Enhanced intrinsic AMY FC network activity in bilateral THA, right insula, and IFG in the study group; Decreased anticorrelated intrinsic AMY FC network activity in left precunesus; The intrinsic AMY FC network activity was positively correlated to impulsivity in right subcallosal gyrus, insula, THA and pCC and negatively correlated in the left fusiform area.
	15 controls (M; 28.9 ± 7.8)	-	9.8 ± 2.9 y	-	-	-	-	
Wang et al., 2016	30 ABS (M; 42.4 ± 5.0);	-	11.1 ± 2.1 y;	7.7 ± 3.6 y	33.9 ± 60.6 w	MMT (52.0 ± 29.4 mg/day)	-	Lower interhemispheric FC between right insula and left inferior OFC, AMY, contralateral insula, putamen and caudate, and lower interhemispheric FC between left insula and right inferior OFC, AMY, contralateral insula, putamen, caudate and rolandic operculum.
	30 controls (M; 41.5 ± 5.2)	-	11.5 ± 1.5 y	-	-	-	-	
Liu et al., 2009	12 ABS (M; 41 ± 5.6);	Yes	8.2 ± 1.8 y;	16.0 ± 3.5 y	25.7 ± 45.4 w	-	-	Stronger connectivity in the PFC (dorsolateral of superior frontal gyrus, orbit part of IFG), aCC, SMA, ventral striatum, insula, AMY and HIPP.
	12 ABS (M; 35.4 ± 7.1)	-	9.1 ± 2.8 y	-	-	-	-	
Ma et al., 2010, 2011	14 ABS (M; 30.1 ± 5.3 y);	Yes	7.1 ± 2.8 y	9.7 ± 2.7 y;	5.6 ± 9.4 w	Mixed groups (12 ABS under MMT)	-	Increased FC in right HIPP; Decreased FC in right dorsal aCC and left caudate; Alterations in FC strength in the mesocorticolimbic system (i.e., increased FC between NAc and ventral and rostral aCC, between NAc and OFC, and between AMY and OFC; and decreased FC between PFC and OFC, and between PFC and aCC).
	13 controls (M; 29.8 ± 7.2 y)	Yes	10.8 ± 1.6 y	-	-	-	-	
Yuan et al., 2010	11 ABS (M; 37.2 ± 7.3 y);	-	9.8 ± 2.5 y;	7.5 ± 4.6 y	21.0 ± 3.4 w	MMT (32.2 ± 18.7 mg/day)	-	Alternation in the rostral aCC network (i.e., reduced FC between rostral aCC and the regions of left OFC, dorsolateral PFC and right middle temporal lobe; reduced FC between precuneus and the regions of the right cerebellum and left dorsolateral PFC); The abuse duration was negatively correlated with the altered rostral aCC network but positively correlated with the degree in the right paraHiPP, left putamen and bilateral cerebellum and yet negatively correlated with the path length in these regions.
	11 controls (M; 36.8 ± 7.4 y)	-	9.1 ± 3.2 y	-	-	-	-	
Zhang et al., 2011	12 ABS (M; 32.7 ± 7.3);	-	9.8 ± 2.5 y;	7.5 ± 4.6 y	147 ± 24 d	MMT (34.2 ± 18.7 mg/day)	-	Significant discrepancy in activation patterns mPFC, OFC, aCC, HIPP, AMY, caudate, putamen, posterior insula and THA between groups.
	13 controls (M; 36.8 ± 7.4)	-	9.1 ± 3.2 y	-	-	-	-	
Zhai et al., 2015	22 ABS (M; 33.1 ± 6.1);	-	10.9 ± 2.4 y;	6.6 ± 3.7 y	56.1 ± 17.5 d	-	-	Stronger network connectivity for the reward system involving in mesocorticolimbic pathway; Weaker network connectivity for executive control system, such as dorsolateral PFC.
	15 controls (M; 28.9 ± 8.1)	-	9.6 ± 0.2 y	-	-	-	-	

*rs, resting state; sd, standard deviation; ABS, opiate abstinent subjects; M, male; X, both genders; y, year; d, day; w, week; MMT, methadone maintenance treatment; FC, functional connectivity; m, medial; p, posterior; a, anterior; PFC, prefrontal cortex; AMY, amygdala; OFC, orbitofrontal cortex; CC, cingulate cortex; IFG, inferior frontal gyrus; HIPP, hippocampus; THA, thalamus; SMA, supplementary motor area; NAc, nucleus accumbens*.

First, alterations in rsFC strength between ventral striatum and various subcortical and cortical regions in the reward system have been observed. In the reward (MCL) circuits, the increased dopamine releases are thought to underlie the initial reinforcing influences of abused drugs (Nestler, 2005). Ma et al. (2010) tested the hypothesis whether or not the MCL circuits were altered in 12 abstinent heroin-using OUD subjects under methadone maintenance treatment (MMT) and 2 abstinent male heroin-using OUD subjects without any treatment and compared with 13 demographic-matched smokers. Results reported enhanced rsFC between the MCL regions and subcortical and cortical areas. In the MCL system, stronger rsFC between nucleus accumben and the ventral areas of medial (PFC), including rostral anterior cingulate cortex (CC) and medial orbitofrontal cortex (OFC) were observed, suggesting that connectivity within reward and motivation circuits may help the interpretation of altered incentive salience for drugs and drug-associated cue. Without considering the smoking habit, the finding of Zhai et al. (2015) was consistent with that of Ma et al. (2010), while Wang et al. (2016), on the other hand, showed reduction in rsFC between MCL regions and subcortical and cortical areas. It was noted that the reported abstinence duration of the OUD subjects underwent MMT (daily dose: 52.0 ± 29.4 mg) in Wang (Wang et al., 2016) study was relatively longer than that of the OUD subjects in Ma (Ma et al., 2010), where the daily dose of MMT was not reported, and that of the OUD subjects in Zhai (Zhai et al., 2015), where the treatment program was not reported in the study. It is not known if these factors (i.e., abstinence length, methadone maintenance length and dosage, and treatment program) played a role in the inconsistency of the rsFC alternation. Thus, more studies are needed to illustrate the hypothesis.

Second, altered connectivity between the circuits associated with emotion and stress regulations, including amygdala and insula, has been observed in OUD patients during multiple stages of dependency. Ma et al. (2010, 2011) reported increased rsFC between amygdala and lateral OFC. Similarly, Liu et al. (2009) reported increased rsFC strength in amygdala, insula, hippocampus, ventral striatum, supplementary motor area (SMA), anterior CC, and inferior frontal gyrus in the PFC in 12 abstinent male heroin–using OUD subjects compared with 12 age-matched controls. These regions were also identified as distinctive to access resting-state brain activities in heroin abstinent OUD subjects (Zhang et al., 2011), and the connectivity strengths in amygdala and insula were also observed in Xie et al. (2011). However, in a sample of chronic heroin–using OUD subjects (*n* = 30) underwent MMT, Wang et al. (2016) observed significant reduction in rsFC between insula and inferior OFC, amygdala, putamen, and caudate areas, where the reduced inter-hemispheric connectivity between these regions may predict a higher incidence of a failure of the integration process—a deviation from the normal neurodevelopmental trajectory (Fair et al., 2007, 2008). Taken together, further investigations in a larger sample (*n* > 30) are required to examine the alternation in the amygdala and insula networks in OUD when working under the assumptions that amygdala and its connected regions are critical neural substrates mediating continued drug intake, and that the neural interactions are essential to process processing emotional stimuli, generating affective states, and regulating emotion (Phillips et al., 2003; Pezawas et al., 2005; Stein et al., 2007).

Third, abnormal connectivity between the neural systems associated with cognitive control brain network, including the anterior CC, lateral PFC and parietal areas, has also been found in abstinent heroin-using OUD subjects. Yuan et al. (2010) found reduced rsFC strength between the left inferior parietal lobe and right dorsolateral PFC in 11 abstinent heroin-using OUD subjects underwent MMT comparing with 11 age-matched controls. Consistent to the hypothesis that rsFC is reduced between the anterior CC and dorsolateral PFC in abstinent heroin-using OUD subjects. Ma et al. (2011) also reported the reduction between the right dorso-anterior CC and left caudate.

## Resting-state electroencephalography (EEG)

The abovementioned cell assembly concept (Hebb, 2005) refers to a mechanism by which a coherent entity through the joint activity of distributed and interconnected neurons exhibits distinct features of a stimulus. In fact, neuronal inhibition plays an essential role in establishing these cell assembly, allowing the precise alignment of the spiking activity of neurons within specific time windows (Singer, 2013). In particular, oscillations between populations of neurons are synchronized by the precisely timed inhibitory signals, and thus fluctuations in neuronal excitability are coordinated (Singer, 1999; Fries et al., 2001; Womelsdorf et al., 2014). Human EEG is a neuroimaging technique that directly monitors and records the electrical activity (i.e., the coordinated oscillatory activity thought to mediate neuronal communication at cellular level) of the cerebral cortex. Scalp EEG activity demonstrates oscillations at different frequencies, categorized in frequency bands. Several of these oscillations have specific spatial distributions and are associated with different states of brain function, such as eye-closed resting state (Knyazeva and Innocenti, 2001). This neuroelectrophysiological approach has been used in resting-state heroin-using OUD studies. Table 2 demonstrates the summary of key findings of resting-state EEG studies in the review.

**Table 2:** **Summary of rs-EEG neuroimaging findings in opiate addiction**.

**Features of samples**								
				**Features of opiate group**				
**Study**	**Sample size (gender; age: mean ± sd)**	**Smoking habit**	**Educational level (mean ± sd)**	**Abuse duration (mean ± sd)**	**Abstinent duration (mean ± sd)**	**Treatment program**	**Concurrent medical patterns**	**Finding**
Fingelkurts et al., 2006a,b	22 ABS (X; 33.0 ± 5.5);	-	-	11.0 ± 5 y	2 w	-	Poly-substance dependence; personality disorder	Increase of local FC and decrease of remote FC (beta, alpha); Temporal and occipital FC in the posterior was not correlated with duration of addiction; In temporal region the composition of brain oscillations percentage and periods of beta activity increased; In occipital, parietal, temporal, and frontal regions the alpha activity increased but the theta activity decreased.
	14 controls (X; 33.0 ± 5.0);	-	-	-	-	-	-	
Fingelkurts et al., 2007	13 ABS (X; 32.0 ± 5.5);	-	-	10.7 ± 6.4 y	2 w	-	Poly-substance dependence; personality disorder	Increased local and remote FC for alpha and beta bands; Increased severity of withdrawal symptoms showed correlation with the percentage of theta-beta and beta bands.
	14 controls (X; 33.0 ± 5.0);	-	-	-	-	-	-	
Fingelkurts et al., 2009	6 ABS (X; 33.0 ± 5.0);	-	-	11.0 ± 7.0 y	11.0 ± 6.0 m	MMT	Poly-substance dependence; personality disorder	Amplitude for increased alpha and increased beta bands in Frontal, Temporal, right parietal (decreased beta), and midline occipital cortex (decreased alpha); Increase of local rsFC and decrease of remote FC (beta, alpha); Temporal and occipital FC in the posterior was not correlated with duration of addiction.
	14 controls (X; 33.0 ± 5.0);	-	-	-	-	-	-	
Gorricho and Usón, 2008	15 ABS (X; 38.9 ± 5.1);	-	- (between primary and secondary education)	16.2 ± 5.2 y	59 ± 33 m	MMT (69.3 ± 17.0 mg/day)	Poly-substance dependence	Abusers underwent MMT showed a decrease in alpha activity and an increase in slow-wave activity (delta and theta bands), suggesting cognitive dysfunction in MMT subjects.
Franken et al., 2004	18 ABS (M; 32.4 ± 5.9)	-	- (between lower and higher education)	9.0 ± 6.3 y	2 w	MMT (10 ABS)	Poly-substance dependence	The measured EEG power and coherence showed increase in beta2 and gamma bands; In the temporal region the beta2 and delta coherence showed correlation with chronic heroin-related thoughts; In the frontal region the alpha1 and the temporal delta coherence showed correlation with chronic heroin craving.
	12 controls (M; 32 ± 9.5)	-	Unmatched (between lower and higher education)	-	-	-	-	

*rs, resting state; sd, standard deviation; ABS, opiate abstinent subjects; M, male; X, both genders; y, year; d, day; w, week; m, month; MMT, methadone maintenance treatment; FC, functional connectivity*.

Fingelkurts et al. (2006a,b, 2007, 2009) examined the local and remote rsFC and the composition of brain oscillations in heroin abusers in longitudinal studies using 20 EEG channels. First, the research group (Fingelkurts et al., 2006a) studied 22 mixed-gender subjects with 4–26 years of heroin-use history who were at the stage of long-term abstinence and underwent MMT, as well as the OUD subjects who were at short-term withdrawal period and compared with 14 demographic-matched controls. Later they (Fingelkurts et al., 2007) focused on the 13 subjects out of the 22 heroin-using OUD subjects who were at the withdrawal period and compared with the 14 controls to examine the differences. It was found that both the long-term abstinent and short-term withdrawal OUD patients demonstrated an increase in local rsFC and temporal stabilization for alpha and beta bands across the cortex (Fingelkurts et al., 2006a), and only the short-term withdrawal OUD subjects displayed an increase in remote rsFC (Fingelkurts et al., 2007). Further, the research group (Fingelkurts et al., 2009) studied 6 abstinent subjects underwent MMT and compared with the same controls. It was discovered that methadone restored local and remote rsFCs in heroin-using OUD subjects. Moreover, the average amplitude of alpha and beta bands increased in the left frontal and right temporal cortices and decreased in the right parietal region. The average length of EEG segments was shorter in the right central for alpha and beta bands, right parietal for beta band, and midline occipital cortex for alpha band. Based on the rs-EEG literature, these longitudinal observations concluded that 6-month MMT moderated distribution of rsFC of alpha and beta bands, normalized the composition of EEG activities, and restored the temporal structure of brain oscillations.

Gorricho and Usón (2008) evaluated the acute effect of methadone among 15 mix-gender subjects underwent chronic MMT, measuring the qEEG before and 3 h after the daily methadone treatment. Results showed that an increase in the slow-wave activity (delta and theta bands) and a decrease in the alpha activity of MMT subjects. Further, Franken (Franken et al., 2004) found that increased relative beta-2 power and increased left intrahemispheric gamma coherence in the temporal and frontal regions in OUD when evaluating EEG power and coherence and brain functionality of 18 male heroin-using OUD subjects and compared with 12 healthy controls. The EEG coherence also showed correlations with clinical measures, such as chronic heroin craving, and having heroin-related thoughts, suggesting that the alterations were methadone-related.

## Neuropsychology: cognitive and behavioral measures

Neuropsychology is a discipline that seeks to understand the interrelationship of a human brain and mind in terms of behavioral science. Neuropsychological testing aims to assess the cognitive functioning of OUD subjects, as well as to determine the nature and extent of possible organic cerebral impairment which may be resulted from cumulative effects of chronic drug use on the central nervous system. By examining the performance patterns, which may characterize and distinguish certain SUD individuals, the neuropsychological assessment or task can serve as a tool to discern functional and clinical relationships between different psychological and cognitive processes, such as memory and reasoning (Miller, 1985). It is traditionally useful in contributing to therapeutic management, as well as in predicting treatment outcome. Table 3 summarizes the main findings of clinical neuropsychological research in OUD.

**Table 3:** **Summary of neuropsychology assessment findings in opiate addiction**.

**Features of samples**									
					**Features of opiate group**				
**Study**	**Measuring task**	**Sample size**	**Smoking habit**	**Educational level (mean ± sd)**	**Abuse duration (mean ± sd)**	**Abstinent duration (mean ± sd)**	**Treatment program**	**Concurrent medical patterns**	**Finding**
**COGNITIVE MEASURES**									
Darke et al., 2000	Controlled Oral Word Association Test; Matrix Reasoning; Complex Figure of Rey; Key Search; Digit Spin Test; Digit Symbol Test; Paired Associates I & II; Wechsler Memory Scale-III; Halying Sentence; Completion; Logical Memory	50 ABS (X; 34.1);	-	10.3 y	13.5 y	3–6 m	-	Alcohol dependence; severe head injury	No difference between the abstinent abusers and controls on any tests; The current abusers with treatment had poorer performance than controls in Halying Sentence Completion, Matrix Reasoning, Digit Symbol, Logical Memory, and Complex Figure Test.
		125 current abusers (X; 38.2);	-	9.8 y	19.4 y	-	MMT; Buprenorphine	Alcohol dependence; severe head injury	
		50 controls (X; 35.8)	-	11.2 y	-	-	-	-	
Mintzer and Stitzer, 2002; Mintzer et al., 2005	Digit symbol substitution test (DSST); Trail-making Task; Two-back working memory task; Word recognition memory; Free recall; Gambling task	20 ABS (X; 40.2 ± 1.8);	Yes	11.2 ± 0.4 y	-	9.0 ± 2.3 m;		Poly-substance dependence	The current abusers underwent MMT displayed cognitive impairment on all the tasks; The abstinent study group performance fall between the current abusers with MMT and control groups; the abstinent abusers showed significantly better performance on DSST on the measures of conceptual flexibility/set-shift time and recognition memory measures but significantly worse than the controls.
		18 current abusers (X; 37.6 ± 1.6);	Yes	11.8 ± 0.3 y	-	-	MMT (67.2 mg/day)	Poly-substance dependence	
		21 controls (X; 34.9 ± 2.0)	-	12.1 ± 0.2 y	-	-	-	-	
Xie et al., 2011	Barratt Impulsive Scale	22 ABS (M; 33.0 ± 6.0);	-	10.9 ± 2.4 y;	6.6 ± 3.7 y	8.0 ± 2.5 w	-	-	Higher impulsivity scores.
		15 controls (M; 28.9 ± 7.8)	-	9.8 ± 2.9 y	-	-	-	-	
Zhai et al., 2015	Barratt Impulsive Scale	22 ABS (M; 33.1 ± 6.1);	-	10.9 ± 2.4 y;	6.6 ± 3.7 y	56.1 ± 17.5 d	-	-	Higher impulsivity scores in terms of attention, motor, and non-planning-indicating higher impulsivity.
		15 controls (M; 28.9 ± 8.1)	-	9.6 ± 0.2 y	-	-	-	-	
Hou et al., 2016	Barratt Impulsivity Scale Ultimatum task	17 ABS (X; 34.6 ± 4.7);	Yes	8.39 ± 2.76 y	10.26 ± 3.52 y	6.4 ± 4.0 m	-	-	Higher scores on Barratt Impulsivity Scale attention/cognitive impulsivity and non-planning impulsivity, but not in motor impulsivity.
		18 controls (X; 35.8 ± 7.6)	Yes	8.14 ± 1.96 y	-	-	-	-	
Passetti et al., 2008	Cambridge Gamble Task; Iowa Gambling Task; Tower of London; Information Sampling Task; Stop-Signal Task; GO/NoGO task	10 ABS (X; 37.7 ± 2.6);	Yes	-	10.2 ± 1.7 y	≥ 30 d	MMT (40.0 ± 3.8 mg);	Poly-substance dependence	There was no significant difference between groups. However, the decision-making gambling tasks were found to predict abstinence from drug at 3 months with high specificity and moderate sensitivity.
		27 current users (X; 37.3 ± 1.5)	Yes	-	15.8 ± 2.0 y	-	MMT (50.0 ± 3.0 mg)	Poly-substance dependence	
Yan et al., 2014	Self-ordered pointing test (sopt); Iowa gambling task (igt); Working memory	58 ABS (35.7 ± 6.4);	Yes	8.4 ± 2.0 y;	8.0 ± 6.5 y	12.6 ± 5.9 m	-	-	Performed poorly on the IGT and SOPT tests than the controls; Working memory impairment was associated with heroin dependence but not with pathology gambling; Years of heroin use were negatively correlated with working memory and affective decision-making performance in the study group.
		60 controls (34.3 ± 8.5)	Yes	9.5 ± 2.6 y	-	-	-	-	
**EMOTIONAL/SOCIAL MEASURE**									
Gerra et al., 2014	State-Trait Anxiety Inventory; Childhood Experience of Care and Abuse Questionnaire; Emotional task	30 ABS (M; 29.0 ± 6.0);	Yes	9.5 ± 0.2 y	5.0 ± 2.2 y	>3 m	Cognitive-behavioral treatment; Group therapy	History of cannabis dependence	Higher level of sensitivity of negative emotions and higher basal anxiety scores; High arousal/anxiety was associated with scores of perceived childhood neglect; Addiction severity was associated to childhood adverse experiences.
		30 controls (M; 28.0 ± 7.4)	-	10.8 ± 0.4 y	-	-		-	
Zhao et al., 2009	Delayed recall of valenced and neutral words Trier Social Stress Test (TSST); Working memory	28 ABS (M; 31.3 ± 4.6);	Yes	9.3 ± 2.1 y	7.5 ± 4.4 y	3.0 ± 1.4 m	-	-	Acute stress at the time of recall enhanced retrieval of positively valenced words but no effect on negative and neutral word retrieval in ABS subjects was observed; No changes were detected for attention and working memory.
		20 controls (30.7 ± 6.8)	Yes	9.7 ± 2.6 y	-	-	-	-	
Zhou et al., 2012	Emotional facial expressions	37 ABS (M; 28.0 ± 3.8);	-	-	-	1–26 m	-	-	Displayed a heightened detection of negative emotion when searching facial stimuli; whereas no difference was found on other two groups; The finding claimed to essentially reflect an emotional bias that accompanied with abstinent heroin abusers.
		20 controls (M; 27.6 ± 4.7);	-	-	-	-	-	-	
		19 psychopathology controls with anxiety/depression disorders (M; 23.1 ± 3.7; inmates)	-	-	-	-	-	-	
Morie et al., 2014	Neutral and emotional GO/NoGO tasks	20 ABS (X; 39.5 ± 10.0);	Yes	12.5 ± 1.5 y	29.0 ± 53.5 m	17.7 ± 26 m	-	Poly-substance dependence	No detectable behavioral (nor electrophysiological) differences in inhibitory responses, and no impulsivity between groups.
		21 controls (X; 41.0 ± 10.0)	-	12.0 ± 2.0 y	-	-	-	-	

*sd, standard deviation; ABS, opiate abstinent subjects; M, male; X, mixed gender; y, year; m, month; w, week; -, either unreported or none*.

Opiate abstinent abusers have been compared to an extensive neuropsychological battery to normal controls (Zhao et al., 2009; Xie et al., 2011; Gerra et al., 2014; Morie et al., 2014; Yan et al., 2014; Zhai et al., 2015; Hou et al., 2016), to patients undergoing treatment program, such as MMT (Darke et al., 2000; Mintzer and Stitzer, 2002; Mintzer et al., 2005; Passetti et al., 2008), and subjects with known mental disorder, such as anxiety and depression (Zhou et al., 2012). These studies yielded some consistent results. First, OUD subjects showed significantly higher impulsivity in terms of attention/cognitive and non-planning, even after opiate use ceases (Xie et al., 2011; Zhai et al., 2015; Hou et al., 2016). Interestingly, when the abstinent study group compared to the control group who were smokers, there was no significant difference in motor impulsivity, whereas there was significance when compared to healthy control.

Second, emotional processes, such as regulating stress and negative emotion, were interrupted after prolonged opiate intake. For example, Zhao et al. (2009) showed that acute stress at the time of recall enhanced retrieval of positively valenced words but no effect on negative and neutral word retrieval in abstinent subjects, suggesting the dysregulation of subjects' emotional memory processing under stress and thus providing insight into designing better treatment for patients with OUD. In addition, both Gerra et al. (2014) and Zhou et al. (2012) showed that abstinent OUD subjects presented highly sensitivity toward negative emotion. Gerra et al. (2014) further showed that the high basal anxiety level in OUD subjects and the severity of drug dependence were highly associated with their adverse childhood experiences.

Third, signs of recovery in cognitive performance were shown after sustained opiate abstinence. For example, when Darke et al. (2000) and Mintzer et al. (Mintzer and Stitzer, 2002; Mintzer et al., 2005) used a variety of neuropsychological tests to assess the cognitive processes of abstinent OUD subjects and compare with both current opioid-using subjects and healthy controls, they found that there was no significant difference between abstinent OUD subjects and healthy controls, and yet the current opioid-using subjects underwent MMT demonstrated significantly poor performance, both studies suggesting that methadone and/or the acute effect of opiate might be associated with cognitive impairment, resulting in long-term opiate consumption. This assumption is also supported by the aforementioned neuroimaging findings (Gorricho and Usón, 2008; Fingelkurts et al., 2009; Ieong, 2013).

## Limitations and future direction

There are several important limitations in the review worth highlighting. First, the current review focuses on the functional connectivity during resting-state on the brain regions in OUD, but there are other forms of brain connectivity, including functional connectivity during task performance and structural connectivity. It remains unknown whether or not the functional connectivity is directly related to cognitive brain functions or the structural connectivity in OUD during abstinence. Future studies may be needed to further investigate and resolve these gaps. Indeed, the relationship between functional network and cognitive function is an essential question that has so far been relatively under-explored (Fornito et al., 2016). Initial work has shown that inter-individual variations in topological properties of functional networks correlated with variability in cognitive performance (Li et al., 2009; Bassett et al., 2011; Zalesky et al., 2011; Crossley et al., 2013, 2014). And thus, in addition to explore brain function network using neuroimaging techniques, exploring the cognitive relevance of the brain areas using neuropsychological testing and observing from clinical symptoms are essential to better understand OUD among SUDs and effectively identify and treat one OUD patient among other patients with SUDs.

Second, although we provide a comprehensive overview of resting-state neuroimaging and neuropsychological studies, the samples sizes are relative small, and the study groups are heterogeneous. It is noted that some of the study groups were with concurrent comorbidity or underwent different treatment programs, such as MMT, buprenorphine maintenance, and group therapy in the review. Although methadone, a full opioid agonist, and buprenorphine, a mixed opioid agonist antagonist, have been the mostly administrated drugs to ease OUD withdrawal symptoms (Schuckit, 2016), both of them bind to the opioid receptors in the brain to produce long-acting effects to relieve symptoms during abstinence when the short-acting opioids, such as heroin and prescription morphine, are discontinued. The current review has not fully addressed the treatment outcomes of these opioids, as well as the possible neural complications that may intervene the brain activities in terms of brain regions, functions and behaviors in OUD. In order to compensate the limitation of heterogeneity and facilitate future studies, the considerably important factors, such as the treatment programs, daily dose of MMT, smoking habit, education levels, and concurrent medical patterns of the subjects reported in the literature were detailed and tabled in the review. Large samples with matched controls should also be considered to confirm the findings and resolve the heterogeneity.

Third, we only briefly explored the social cognitive processes in OUD subjects because empirical studies are limited. Living in a complex social environment, one's decision-making often takes place in a social setting and is influenced by one's social cognitive abilities (Bargh, 1989) and personal experiences from childhood to adulthood (Chapman et al., 2004). To date, socio-cognitive process has not been fully investigated in an experimental research setting in OUD. We suggest the use of the powerful tools in neuroimaging and neuropsychology to further explore the chronic effect of opiate on cognitive and emotional processes in a social interactive environment or setting.

Further, in addition to the relatively high-cost fMRI to measure the hemodynamic responses, future studies can consider the alternative, non-invasive, rapid-growing and cost-effective functional near-infrared spectroscopy (fNIRS) as a neuroimaging tool to study functional connectivity in OUD (Ieong, 2015).

## Conclusion

Recovery requires more than abstinence in OUD. Maintaining abstinence may be considered one step toward long-term recovery. The primary aim of the review was to present the current and comprehensive key findings using neuroimaging and clinical neuropsychological tools to explore the chronic influence of opiate during abstinence. We summarized that OUD subjects who stayed abstinence for 2 weeks or above demonstrated: (1) strengthened brain connectivity in the reward system and enhanced beta and alpha power activity, (2) recovery signs in several cognitive and behavioral performances but not in impulsivity, and (3) normalization of brain oscillations in the temporal area induced by methadone treatment in approximately 6 months and yet showed no cognitive improvement by methadone.

Given the current advances in assessing the brain network through rsFC generated from the times series of measured neurophysiological data (i.e., changes in cerebral blood flow, electrophysiological signals on scalp), the powerful neuroimaging tools in the review have high potentials to identify potential biomarkers and to generate both system-level and cell-level network topologies in OUD. Further, the traditional neuropsychological assessment tools have provided evidence of the change in certain brain functions and behaviors during abstinence in OUD. Considering that dysregulation of emotional processes and hyper-sensitivity to negative emotions in OUD patients even after prolonged abstinence, understanding the associations between maladaptive social interaction and the withdrawal syndromes in OUD are fundamentally important. After long-term cessation of opiates, withdrawal syndromes including generalized pain and discomfort and the psychological symptoms, such as restlessness and anxiety, start to merge and may stay from weeks to years (Schuckit, 2016). In addition to these distress during abstinence and social discrimination in the environment, it is not difficult to predict a relapse in an OUD abstinent patient whose reward MCL circuits and emotional mediating system have been disrupted resulting from chronic and intense opiate use—if an efficient diagnostic, a positive intervention and effective treatment programs fall apart.

In sum, when we address some limitations, we hope this review using multidisciplinary approach will provide a useful conceptual model of evaluating interventions and will shed light on the brain regional, functional and behavioral domains of OUD.

## Author contributions

HI performed the literature review, wrote and contributed intellectually to the paper. HI and ZY read and approved the final manuscript.

## Funding

This article was supported by grants MYRG2014-00093-FHS, MYRG 2015-00036-FHS, and MYRG2016-00110-FHS from the University of Macau in Macao SAR and grants FDCT 026/2014/A1 and FDCT 025/2015/A1 from the Macao government.

### Conflict of interest statement

The authors declare that the research was conducted in the absence of any commercial or financial relationships that could be construed as a potential conflict of interest.
